# Supplementary figures and images for: Variable Intrinsic Expression of Immunoregulatory Biomarkers in Breast Cancer Cell Lines, Mammospheres, and Co-Cultures
Source: Int J Mol Sci. 2023 Feb 24;24(5):4478. doi: 10.3390/ijms24054478 (PMC10003642; doi:10.3390/ijms24054478)

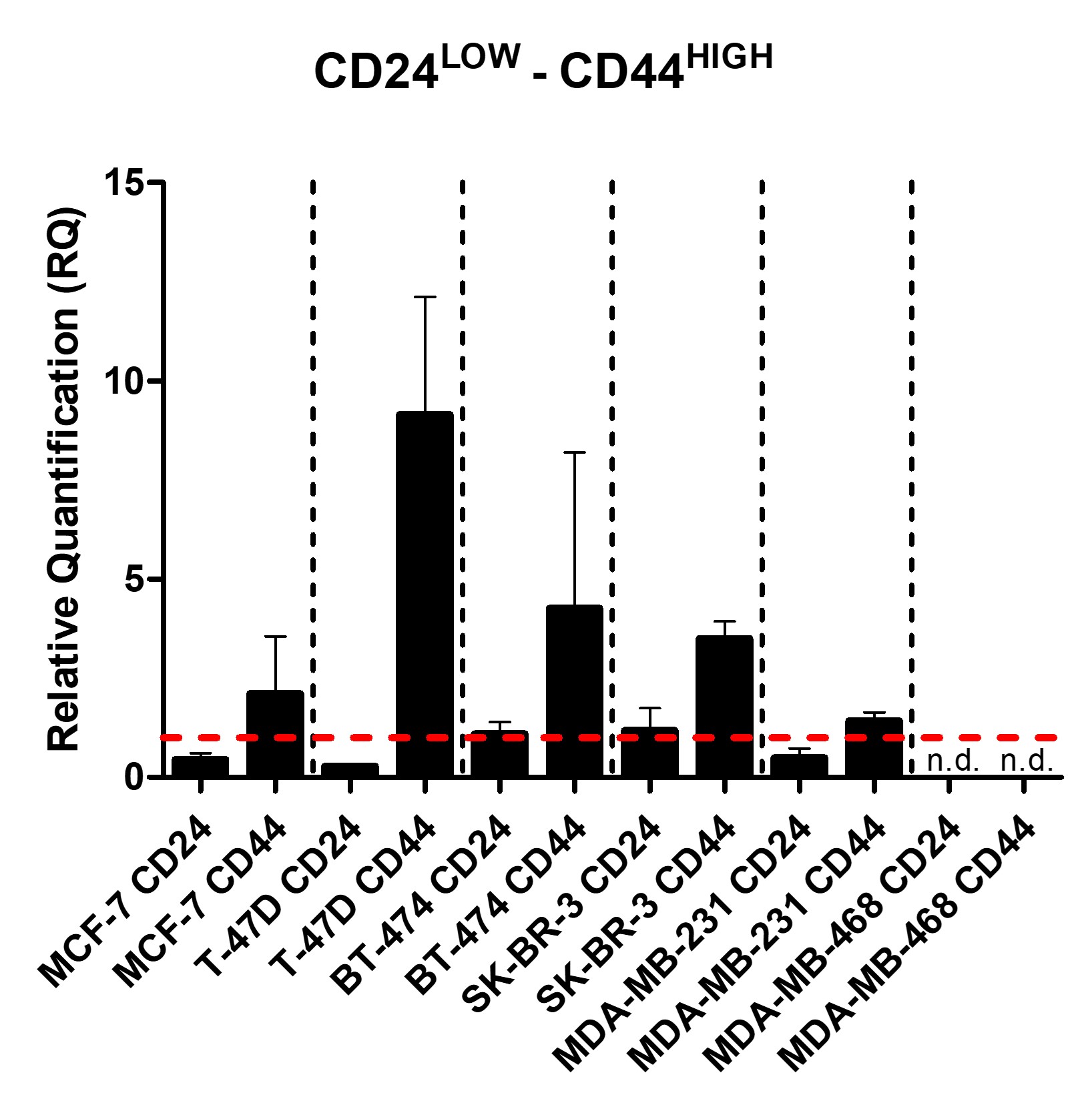

Supplement: Supplementary file 1 [file ijms-24-04478-s001.zip › ijms-2094978-supplementary.jpg]
